# Selection of Potent Non-Toxic Inhibitory Sequences from a Randomized HIV-1 Specific Lentiviral Short Hairpin RNA Library

**DOI:** 10.1371/journal.pone.0013172

**Published:** 2010-10-08

**Authors:** Carola Pongratz, Benjamin Yazdanpanah, Hamid Kashkar, Maik J. Lehmann, Hans-Georg Kräusslich, Martin Krönke

**Affiliations:** 1 Institute for Medical Microbiology, Immunology and Hygiene, University Hospital Cologne, Cologne, Germany; 2 Cologne Excellence Cluster on Cellular Stress Responses in Aging Associated Diseases (CECAD) at the Institute for Genetics, University of Cologne, Cologne, Germany; 3 Center for Molecular Medicine at the University of Cologne, Cologne, Germany; 4 Department of Virology, University Hospital Heidelberg, Heidelberg, Germany; University Hospital Zurich, Switzerland

## Abstract

RNA interference (RNAi) has been considered as an efficient therapeutic approach against the human immunodeficiency virus type 1 (HIV-1). However, to establish a durable inhibition of HIV-1, multiple effective short hairpin RNAs (shRNAs) need to be stably expressed to prevent the emergence of viral escape variants. In this study, we engineered a randomized lentiviral H1-promoter driven shRNA-library against the viral genome. Potent HIV-1 specific shRNAs were selected by ganciclovir treatment of cell lines stably expressing the cDNA of Herpes Simplex Virus thymidine kinase (HSV-TK) fused to HIV-1 nucleotide sequences. More than 50% of 200 selected shRNAs inhibited an HIV-1 based luciferase reporter assay by more than 70%. Stable expression of some of those shRNAs in an HIV-1 permissive HeLa cell line inhibited infection of wild-type HIV-1 by more than 90%. The combination of a randomized shRNA-library directed against HIV-1 with a live cell selection procedure yielded non-toxic and highly efficient HIV-1 specific inhibitory sequences that could serve as valuable candidates for gene therapy studies.

## Introduction

RNAi is a highly conserved process that protects the host from transposable elements and viruses [1], [2]. In mammalian cells, RNAi can be initiated by the introduction of synthetic double stranded short interfering RNAs (siRNAs) of 19–21 basepairs, which are recognized by the cytoplasmic RNA induced silencing complex (RISC) and trigger the specific degradation of complementary mRNA, thereby circumventing an interferon response [3]. Besides its role as an experimental technique for loss-of-function studies [4], [5], RNAi emerges as a powerful therapeutic approach towards human diseases as well as viral infections [6], [7]. Different vectors have been developed both for transient and stable expression of the inhibitory RNA species [8], more commonly by RNA Polymerase III driven transcription of the inverted sense and antisense strands of shRNAs, connected by a linker-sequence (loop) [9], [10]. In the case of HIV-1, siRNA- and shRNA-mediated post-transcriptional gene silencing offers a promising strategy to inhibit viral replication at different stages of viral infection by targeting viral as well as host genes [11], [12], [13], [14]. However, the stable expression of antiviral shRNAs did not evoke the expected long-term inhibition of the virus due to the emergence of viral escape mutants [15], [16], [17]. To circumvent the emergence of RNAi-resistant variants it seems to be mandatory to identify multiple potent inhibitory sequences targeting different or conserved regions of the viral genome [18], [19], [20], [21].

There are several publicly available design algorithms [22], [23], but reliable rules for efficient siRNA-sequences are still missing and designed siRNAs need to be assayed for their inhibitory potential and possible cytotoxic or other off-target effects [24]. Furthermore, the costly and time-consuming synthesis and validation of designed siRNAs limit their use in large-scale studies. Consequently, several groups have developed techniques for the generation of randomly prepared shRNA libraries [25], [26], [27]. All these approaches employ a similar principle for the construction of the library: cDNA is enzymatically converted to short inverted repeats separated by a linker-sequence and transcribed into shRNAs by appropriate expression systems. However, the feasibility and efficiency of these techniques has been disputed [28].

We have adopted and modified these technologies to generate the first randomized shRNA-library against the HIV-1 genome. A stringent cell-based selection procedure was introduced, which allowed for the identification of novel and potent HIV-1 specific shRNAs with little or no host cell toxicity. Moreover, a range of these virus-specific shRNAs were applied as synthetic siRNAs with a comparable efficacy. Additionally, the same sequences were stably expressed in permissive HeLa CD4 (HeLa P4) cell lines [29], where they effectively inhibited wild-type HIV-1 infections. Thus, our data demonstrate for the first time that a straightforward shRNA-library construction protocol in line with a stringent cellular selection procedure yield potent and readily convertible shRNAs against a viral genome.

## Materials and Methods

### Construction of the shRNA-library

The construction of the shRNA-library can be divided into 7 steps. **Step 1:** 5 µg viral DNA (pNL4.3; NIH AIDS Research and Reference Reagent Program) was partially digested with 1×10^−3^ U DNaseI in the presence of 1 mM MnCl_2_ (10′, 37°C). DNaseI was inactivated by vortexing (30″) and heat-inactivation (10′, 65°C). Cleaved 5′ and 3′ ends were repaired with 5 U T4 DNA Polymerase and 10 U Klenow Fragment in the presence of 200 µM dNTPs. DNA was separated on 20% polyacrylamid in TBE and fragments of the appropriate size (150–300 bp) were isolated by diffusion in 0.5 M NaCl; 1 mM EDTA (1h, 50°C, shaking) and purified by EtOH precipitation. **Step 2:** 4.5 µg of the DNA was ligated to 1.5 µg of the 3′loop. Subsequently the T4 ligase was heat-inactivated (10′, 65°C) and the DNA was digested with MmeI (1h, 37°C). The reaction was separated on 20% polyacrylamid in TBE and the product of 3′ loop and gene fragment was isolated and purified. The PAGE-purified 3′ loop (5′-GTTGAATCCCGGTTCAAGAGACCGGGATCCAAC) was purchased from QIAGEN (Hilden, Germany) and contained a MmeI restriction enzyme site. **Step 3:** 1 µg of the purified DNA fragment was ligated to 500 ng 5′loop. The reaction was separated on 20% polyacrylamid in TBE and the single-stranded circular DNA ring of 3′ loop, gene fragment and 5′ loop was isolated and purified. The PAGE-purified 5′loop (5′-GGAGAGACTCACTGGCCGTCGTTTTACCAGTGAAGATCTCCNN) was purchased from Qiagen. **Step 4:** The purified DNA ring was used for a RCA using 10 U Φ29 Polymerase and the 5′loop specific primers RCA1 (5′-ACTGGTAA) and RCA2 (5′-GCCGTCGT) in the presence of 0.01 U Pyrophosphatase and 200 µM dNTPs (o.n., 30°C). The RCA products were digested with BglII and MlyI (1h, 37°C) and separated on 20% polyacrylamid in TBE. The palindromic, inverted repeats were isolated and purified. **Step 5:** 10 ng of the BglII-MlyI – digested fragments were ligated to 100 ng of pENTR/shLib. The transformed bacterial colonies were pooled and the primary shRNA-library isolated. **Step 6:** The isolated plasmids were digested with BamHI and re-ligated (pENTR/shLib -BamHI). The resulting bacterial colonies were pooled as the secondary shRNA-library. **Step 7:** The shRNA-expression cassettes were subcloned into pLPac/shLib (LR Clonase™ II Enzyme Mix, Invitrogen, Karlsruhe, Germany).

### Generation of pENTR/siLib

pENTR/ siH1/mDD-stuffer [30] was linearized with BbsI and the resulting 5′ T-overhangs were blunt-ended using 5 U T4 DNA Polymerase in the presence of 200 µM dATP. After digestion with BglII, shRNA-sequences were ligated between the H1-Promoter and the polyT cassette.

### Generation of pENTR/TA

2 inverted Xcm I restriction enzyme sites were created by annealing 36 nt single-stranded oligonucleotides with 5′BamHI and 3′HindIII overhangs (MWG, sequences on request) and ligated into pENTR/siH1/mDD-stuffer. Digestion of the pENTR/TA with XcmI allows for TA-cloning of the Taq-Polymerase amplified shRNA-expression cassettes.

### Generation of pLP/EGFP/siLib

The puromycin-gene was PCR-amplified from pPGK-puro (K. Rajewski, Harvard medical school; primer-sequences on request). The PCR-product was digested with SmaI and Bpu1102I and ligated into the GATEWAY® compatible lentiviral vector pL [30].

### Generation of selection constructs

The HSV-TK gene was PCR-amplified from pRapid-flirt (A. Waisman, Medical Hospital Mainz, [30]) and ligated into pENTR/siH1/mDD-stuffer to yield pENTR/TK. Afterwards, three overlapping HIV-1 genomic segments were PCR-amplified and ligated between HSV-TK ORF and the polyA to yield the selection constructs pENTR/TK/HIV-Lib1, pENTR/TK/HIV-Lib2 and pENTR/TK/HIV-Lib3, respectively (primer-sequences on request). The selection cassettes were next subcloned into the lentiviral vector pL by means of LR Clonase™ II Enzyme Mix (Invitrogen, Karlsruhe, Germany).

### Production of lentiviral particles

The production of lentiviral particles was performed as described in the Manual ‘ViraPower™ Lentiviral Expression System’ (Invitrogen, Karlsruhe, Germany).

### Generation of stable cell lines

HeLa wt or HeLa P4 cells were transduced with viral particles at a (MOI: 0.1) in the presence of 6 µg/ml polybrene. 48h post transduction, cells were treated with 10 µg/ml blasticidin for 10 days. Surviving clones were expanded in 3 µg/ml blasticidin and analysed for stable integration of the transgene and expression of HSV-TK protein and the fusion-mRNAs.

### Selection of efficient HIV-1 specific shRNAs

The library selection cell lines HeLa^TK/HIV-LIB1^, HeLa^TK/HIV-LIB2^ and HeLa^TK/HIV-LIB3^ were transduced with lentiviral particles of the relevant shRNA-libraries (MOI: 0.1). 48h post transduction, selection cell lines were treated two days with 1µg/ml puromycin followed by ganciclovir treatment (2×10^−6^ M) for 5 days, replacing the selection media each day. Surviving cells were expanded clonally and the integrated shRNAs were recovered by nested PCR (primer sequences on request).

### Western blot analysis

Equal amounts of proteins were probed either with HSV-TK antibody (W.C. Summers, Yale University), HIV-1 Integrase antibody (acris antibodies, Herford, Germany), Hsp70 antibody (BD Pharmingen, Heidelberg, Germany) or β-actin antibody (Sigma-Aldrich, Muenchen, Germany), and with horse-radish peroxidase-conjugated antibody to rabbit or mouse IgG (Sigma-Aldrich, Muenchen, Germany) as the primary and secondary antibodies in blocking buffer (10% milk in TBS), respectively.

### mRNA isolation and Northern Blot analysis

Total RNA was isolated using TRIZOL® (Invitrogen, Karlsruhe, Germany). Enrichment of mRNA was achieved with the NucleoTrap mRNA kit (Macherey-Nagel, Düren, Germany). 1 µg mRNA was separated on denaturing 1.8% Agarose in MOPS buffer and transferred onto positively charged nylon membranes in 20×SSC. Membranes were pre-hybridized (4h, 65°C) and hybridized with ^32^P-labeled HSV-TK cDNA probe (overnight, 65°C). Washed membranes were exposed to Phosphoimager screens.

### Luciferase activity assays

HEK 293 FT cells (Invitrogen, Karlsruhe, Germany) were co-transfected with either PCR-amplified shRNA-expression cassettes or shRNA-expressing vectors and pNL4.3Luc.R-E- (NIH AIDS Research and Reference Reagent Program). Luciferase expression was measured 48h post transfection using the Luciferase Assay Reporter Kit (Promega, Mannheim, Germany) and normalized to total protein.

### Sequencing

The PCR amplified shRNA expression cassettes were cloned into pENTR/TA and sequenced via TouchDown-PCRs using the BigDye® Terminator v3.1 Cycle Sequencing Kit in combination with the dGTP BigDye® Terminator v3.1 Cycle Sequencing Kit (both applied biosystems) in a ratio of 3∶1 and in the presence of 0.83 M Betaine.

### IFN-β and P24 antigen ELISAs

IFN-β ELISAs (PBL Biomedical Laboratories, Piscataway, NJ) and p24 ELISAs (Aalto Bio Reagents Ltd., Dublin, Ireland) were performed as described by manufacturers instructions.

### Detection of cell death via crystal violet staining

3×10^3^ cells were seeded overnight per well of a 96 well plate. Adherent cells were quantified with crystal violet dye as previously described [30] for up to 5 days and normalized to day 1.

## Results

### Construction of a randomized shRNA-library targeting the HIV-1 genome

**Figure 1:**
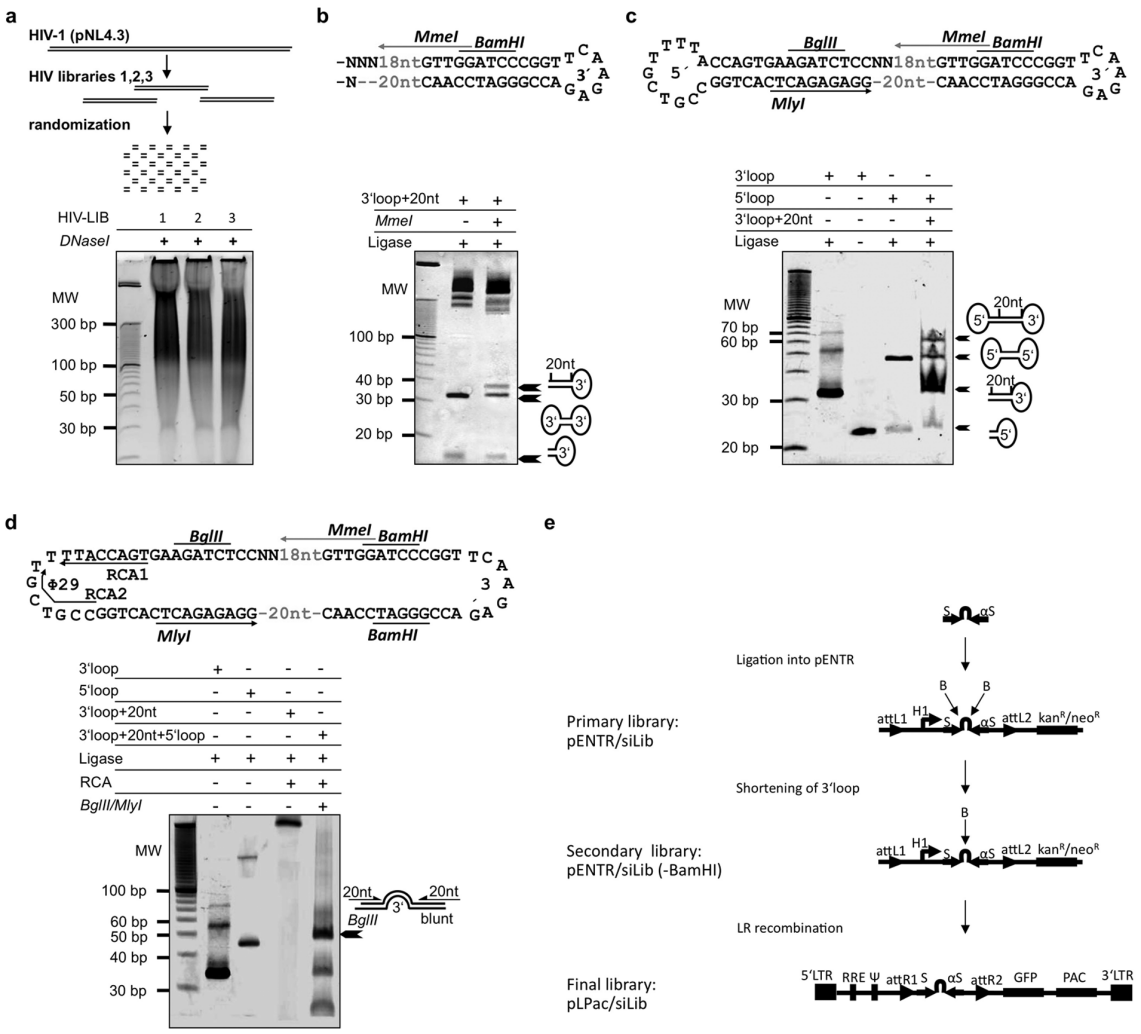
Construction of the shRNA-library. **a**) The 3 HIV segments of pNL4.3 were fragmented using DNaseI and blunt-ended. **b**) Fragments of 150–300 bp were eluted and ligated to the 3′ loop. To limit the size of the HIV-1 inverted repeats, a recognition site for MmeI, which cleaves exactly 20nt from its recognition site and leaves 2 nt 5′ overhangs, was engineered into the 3′ loop. **c**) Ligation of the 5′ loop to the MmeI-digested fragments generated a quasi-circular single-stranded structure. **d**) Rolling circle amplification (RCA) reactions using Φ 29 DNA polymerase and the primers RCA1 and RCA2 were performed to amplify the single-stranded circular DNA and to generate the complementary strand yielding a DNA concatemere of palindromic, inverted repeats encoding siRNA molecules. Digestion with BglII and MlyI liberated shRNA sequences which were inserted into the expression vector pENTR/siLib. **d**) The shRNA sequences were cloned into the linearized pENTR/Lib generating the primary library which was religated after BamHI digestion yielding the secondary library. The final lentiviral shRNA-library was generated by LR recombination of the secondary library into pL/EGP/siLib.

The construction of the randomized lentiviral shRNA-library against HIV-1 was based on a previously described technology with some modifications [26]. The consecutive enzymatic steps to produce the shRNA-library based on the cDNA of HIV-1 are shown in Figure 1 and are described in detail in the Material and Method section. In order to circumvent the production of wild-type virus in selection cell lines, the HIV-1 genome (pNL4.3) was divided into three internal overlapping segments, comprising all genes of HIV-1. The three parts HIV-1.1 (2.5 kb), HIV-1.2 (2.5 kb) and HIV-1.3 (2.8 kb) comprised the NL4.3 nucleotides 855–3374, 3297–5796 and 6067–8447, respectively. In order to maximize the diversity of the shRNA-library, the HIV-1 segments were randomly fragmented with DNase I (Fig. 1a) which exhibits only minor if any sequence specificity [31], [32]. Blunted fragments of about 150–300 bp were ligated to the 3′loop which connects the final sense and antisense strands and enables the restriction of the target sequence to 20 basepairs by the insertion of the Mme I recognition site at the start of its stem (Fig. 1b). The subsequent ligation of the 5′loop produces single-stranded DNA rings which were amplified by rolling circle amplification (RCA) to concatemers of inverted repeats that are flanked by Bgl II and Mly I restriction sites (Fig. 1c–d). The resulting palindromic, inverted repeats encoding shRNA molecules were inserted downstream of the RNA Polymerase III Promoter H1 derived from pSUPER [33] and upstream of the poly-T termination site of the GATEWAY® compatible linearized vector pENTR/shLib (Fig. 1e, primary library). Excess 3′ loops were removed by digestion with Bam HI. The following religation yielded expression-ready shRNA-vectors (Fig. 1e, secondary library). To generate the final HIV-1 shRNA-library, the shRNA-sequences were subcloned by LR recombination into the lentiviral vector pLP/EGFP/shLib (Fig. 1e, final library) which allows for the enrichment of transduced cell lines by puromycin selection and fluorescence monitoring by means of enhanced green fluorescence protein (EGFP).

The combined complexity of the primary libraries was about 1.4×10^4^ (1750 colonies per kB of cDNA). Sequencing of 10 individual clones revealed 10 different HIV-1 specific shRNA-sequences consisting of sense and antisense strand as well as the loop sequence (not shown). Thus, our modifications allowed us to elevate the complexity of our shRNA-library as compared to the original technology, which utilized restriction enzymes for fragmentation of target DNA. Furthermore, we amplified the number of colonies by a factor of about 10 in order to maintain the complexity of the libraries throughout the subsequent cloning steps (Table 1). Hence, our data suggest that the randomization of a cDNA by DNaseI produces a broader diversity and complexity of a given library [26].

**Table 1:** Clone size of randomized shRNA-libraries.

	primary library pENTR/siLib	secondary library pENTR/siLib	final library pL/EGFP/siLib
library 1	4.5×10^3^ cfu	1.6×10^4^ cfu	>10^5^ cfu
library 2	3.4×10^3^ cfu	1.4×10^4^ cfu	>10^5^ cfu
library 3	6×10^3^ cfu	1.5×10^4^ cfu	>10^5^ cfu

To indicate the complexity of the shRNA library, the number of bacterial colonies of each transformation step (primary library, secondary library and final library) was documented as colony forming units (cfu).

### Generation and validation of library selection cell lines

**Figure 2:**
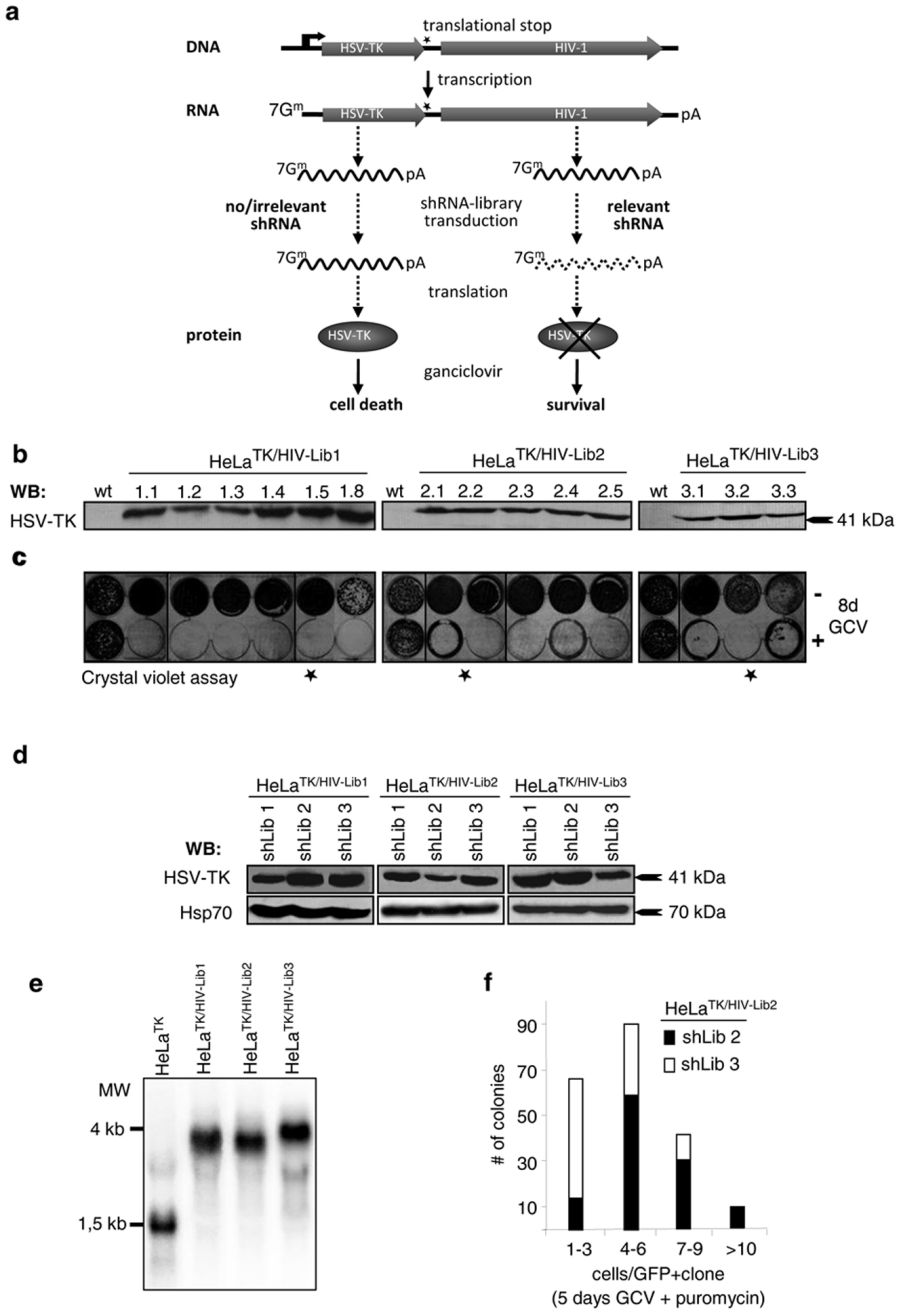
Generation of HIV-1 specific selection cell lines. **a**) HeLa selection cell lines stably express a fusion-mRNA consisting of the HSV-TK upstream of a untranslated fragment of the HIV-1 genome (pNL4.3), that acts as target mRNA for the shRNA-library. Only cells which express an efficient HIV-1 directed shRNA do not express HSV-TK and are able to proliferate. **b**) Western blot analysis of stably transduced HeLa selection cell lines with a HSV-TK specific antibody. Parental HeLa cells were used as control. **c**) Selection cell lines and HeLa wt cells were functionally analysed by 8 days of ganciclovir (GCV: 2×10^−5^ M) treatment. Surviving cells were stained with crystal violet. Designated clones (*) were used to select the shRNA-library. **d**) Western blot analysis of HSV-TK expression in HeLa selection cell lines transiently transfected with pooled shRNA-libraries. Only relevant libraries downregulate HSV-TK in the corresponding selection cell line. **e**) Expression of the fusion-mRNA was examined by Northern blot analysis with a HSV-TK specific probe. **f**) Transduction and subsequent puromycin (2 days) and GCV-selection (5 days) of a relevant (shLib2) and not of an irrelevant shRNA-library (shLib3) allows for clonal expansion of transduced cells.

In order to maximize the stringency of the selection scheme, our method employed a fourfold combination of negative/positive selection measures, comprising ganciclovir (negative) [27] and blasticidin selection of stable selection cell lines (Fig. 1a) in line with selection of the shRNA library via puromycin, and EGFP fluorescence (positive), respectively. Several stably transduced HeLa selection clones (HeLa^TK/HIV-LIB1^, HeLa^TK/HIV-LIB2^ and HeLa^TK/HIV-LIB3^) were shown to express the HSV-TK protein (Fig. 2b) and to be functional by means of ganciclovir induced cell death, as visualised by crystal violet staining of adhering cells (Fig. 2c). Furthermore, we tested if our pooled lentiviral shRNA library is able to downregulate the HSV-TK expression in the corresponding selection cell lines. Upon transient transfection of the shRNA libraries into the selection cell line clones indicated by asterisks in Fig. 2c, only the complementary library is capable of downregulating the HSV-TK expression significantly as compared to the non-matching libraries (Fig. 2d).

Furthermore, the proper expression of the fusion-mRNAs was verified by Northern blot analysis of mRNA from these selection cell lines using a HSV-TK specific probe (Fig. 2e). Finally, we show that in the presence of puromycin and gancyclovir only the transduction of a relevant shRNA-library into the corresponding selection cell line enables the transduced cells to proliferate and form colonies, suggesting that our selection design represents a highly stringent procedure for the selection of shRNA-libraries (Fig. 2f).

### Selection of potent HIV-1 specific shRNAs

**Figure 3:**
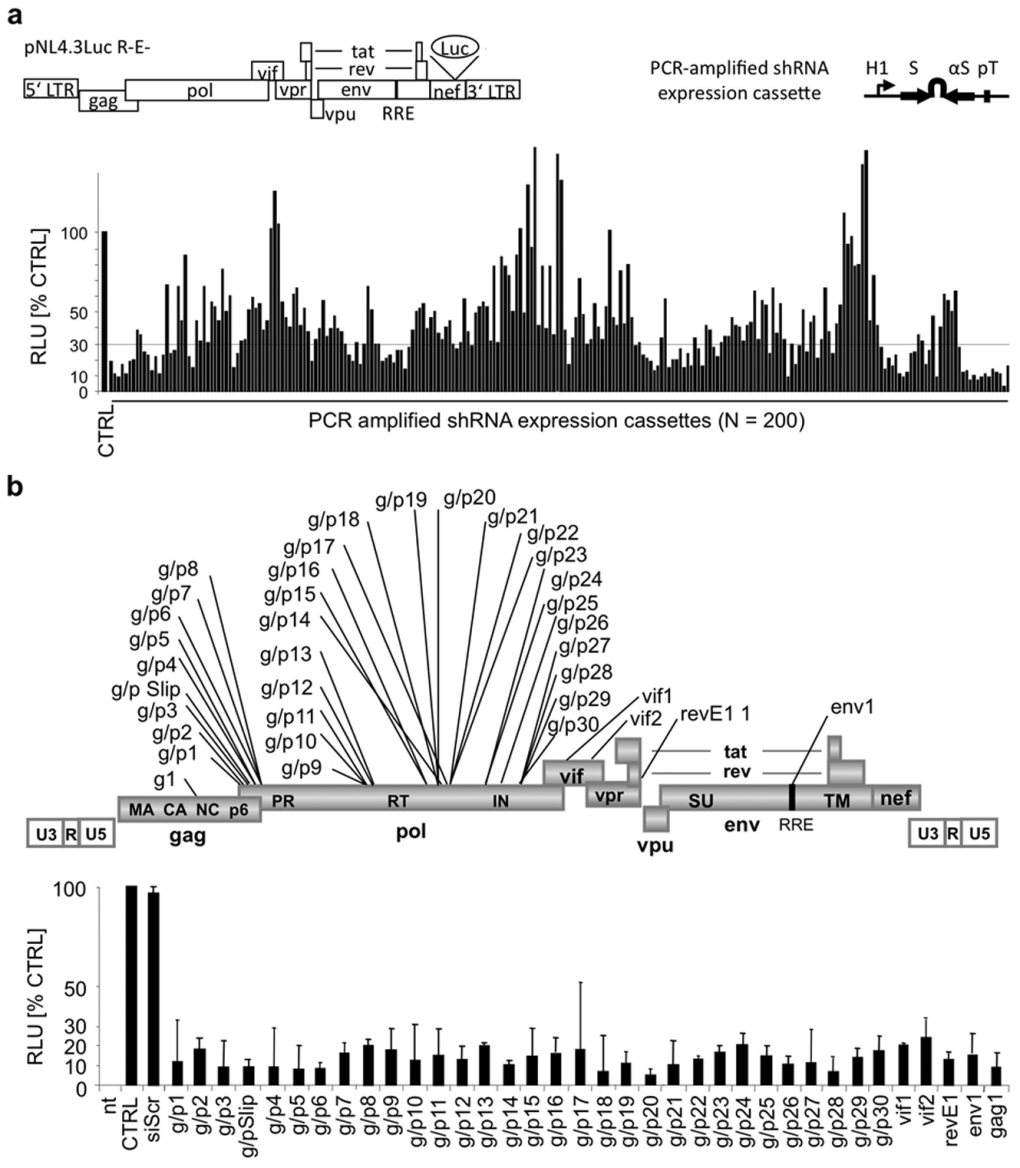
Reconfirmation of the inhibitory potential of selected shRNA-sequences. **a**) ShRNAs were PCR-amplified from genomic DNA of selected cell clones as shRNA expression cassettes consisting of H1 promoter, shRNA-sequence and polyT. Each of the 200 individual cassettes were co-transfected with the HIV-1 specific Luciferase reporter construct pNL4.3Luc.R-E- into HEK 293 FT cells. Luciferase expression was measured 48 h p.t. Cells transfected with pNL4.3Luc.R-E- and a scrambled shRNA (sh scr) expressing cassette were used as control. **b**) Map of the identified shRNAs and re-evaluation of their inhibitory potential upon co-transfection as described in **a**). Cells which were transfected with pNL4.3LucR-E- alone or co-transfected with a scrambled siRNA control served as controls. Error bars indicate +/− SD of mean of three independent experiments.

The lentiviral shRNA-libraries were stably transduced into the corresponding selection cell lines and treated with ganciclovir in order to select for potent shRNAs. Transduced cells were then treated with puromycin and only surviving, EGFP positive colonies were picked for analysis. Next, shRNAs were recovered by nested PCR amplification of shRNA-expression cassettes [34] from the genomic DNA of surviving cell clones. PCR amplified shRNA cassettes were subjected to co-transfections into HEK 293 FT cells along with the HIV-1 specific luciferase reporter construct pNL4.3luc.R-E-. Of the 200 individual cassettes tested, more than 50% inhibited luciferase activity by over 70% as measured 48h post transfection and are thus considered as effective (Fig. 3a).

To enable a vector-based expression of those shRNA-sequences we subcloned a collection of highly potent PCR-amplified shRNA-expression cassettes into the GATEWAY® compatible TA-cloning vector pENTR/TA. 36 recovered shRNA-expression cassettes were sequenced and their inhibitory potential was reconfirmed in the HIV-1 specific luciferase assay described above (Fig. 3b). The relative nucleotide positions and HIV-1 target sequences of this selection are depicted in Figure 3b and Table 2. 24 individual shRNAs were directed against the *gag* (g) and *gag-pol* transcript (g/p), 2 shRNAs against vif (vif), one against rev (revE1) and one against env (env), respectively. Subsequent BLAST analysis against the HIV-sequence data base of the *Los Alamos National Laboratory* (http://www.hiv.lanl.gov) revealed that some shRNAs target well conserved HIV-1 sequences, whereas some target more variable regions. It is worth emphasizing that none of our shRNA-sequences has been described so far, pushing us to analyse the selected sequences using RNAi design algorithms [24]. Interestingly, none of our sequences matched all parameters of those algorithms, suggesting that there are still unknown sequence characteristics for potent siRNAs and shRNAs.

**Table 2:** Specificity of shRNAs against HIV-1.

shRNA	target sequence (pNL4.3)	Starting nt	Los Alamos hits
g 1	agctaccataatgatacaga	1454	7288
g/p 1	tcagagcagaccagagccaa	1642	8767
g/p 2	ccaccagaagagagcttcag	1668	9767
g/p 3	gagacaacaactccctctca	1699	35180
g/p Slip	ctgagagacaggctaattt	1722	45201
g/p 4	gacaacaactccctctcaga	1749	10211
g/p 5	acaactccctctcagaagca	1753	3583
g/p 6	acagcgacccctcgtcacaa	1771	10267
g/p 7	tctgagagggagttgttgtc	1816	65399
g/p 8	tacaggagcagatgatacag	1873	66221
g/p 9	gtacagcctatagtgctgcc	2816	43997
g/p 10	acagctggactgtcaatgac	2822	34735
g/p 11	gtcaatgacatacagaaatt	2855	35518
g/p 12	ggcaagtcagatttatgcag	2893	33484
g/p 13	gaagcagagctagaactggc	2987	27153
g/p 14	acagagtattggcaagcaac	3302	60013
g/p 15	agagtattggcaagccacct	3304	3986
g/p 17	gagtgggagtttgtcaatac	3322	3579
g/p 18	gtcaatacccctcccttagt	3344	3456
g/p 19	actttctatgtagatggggc	3410	2931
g/p 20	aaagttgtccccctaacgga	3487	25962
g/p 22	ccagcacacaaaggaatt	4114	2825
g/p 23	ggacaagtagactgtagccc	4384	2554
g/p 24	agtactacagttaaggccgc	4142	30137
g/p 26	gtcaaggagtaatagaatct	4215	3121
vif 1	tggttttatagacatcacta	4698	3034
vif 2	gaacaagccccagaagacca	5108	4591
rev E1	gagctcatcagaacagtcag	5545	4545
env 1	tccaggcaagaatcctggct	7487	12141

**a)** Designation of the identified shRNAs **b)** shRNA-target sequence of the HIV-1 proviral clone pNL4.3 **c)** starting nucleotide of the shRNA based on +1 being the transcriptional start site. **d)** Number of known isolates which include the target sequence.

These data suggest that our stringent fourfold selection protocol yielded a high proportion of potent inhibitory shRNAs that are unique in their sequence preferences as compared to those predicted by publicly available design algorithms.

### Antiviral activity and cellular tolerance of siRNA and lentiviral shRNA derivatives

**Figure 4:**
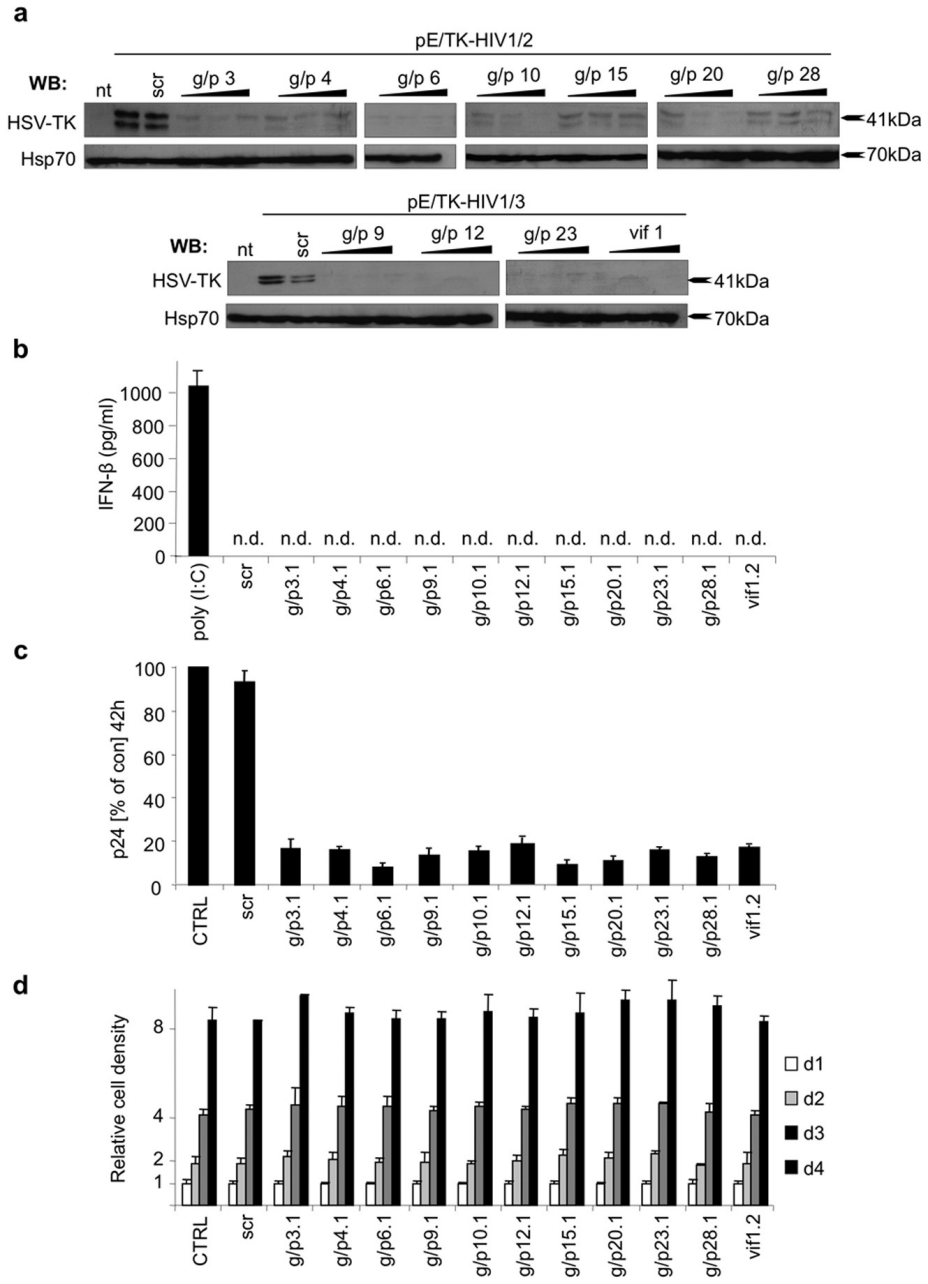
Convertibility of the shRNA-library species and cellular tolerance of siRNA and lentiviral shRNA derivatives. **a**) 11 individual shRNAs were chemically synthesized as siRNAs and co-transfected (5 nM, 20 nM and 100 nM) with the corresponding HSV-TK selection construct into HEK 293 FT cells. Western blot analysis with a HSV-TK specific antibody 48h p.t. revealed that the synthetic siRNAs efficiently inhibit HSV-TK expression in a dose-dependent manner. Hsp70 was used as loading control. **b**) IFN-β ELISAs were performed with HeLa cells transfected with siRNAs or poly (I∶C). None of the siRNAs induced any detectable amounts of IFN-β. **c**) The same sequences were stably expressed as lentiviral shRNAs in the HIV-1 permissive HeLa P4 cells. Individual clones were infected with HIV-1 and the production of p24 was determined via ELISA 42 h p.i.. **d**) The viability of the clones was determined at the indicated time points using crystal violet, demonstrating no detrimental effects of shRNA expression. Error bars indicate +/− SD of mean of three independent experiments.

In eukaryotic cells, shRNAs are transcribed in the nucleus and exported to the cytoplasm by exportin-5 where they are processed into functional siRNAs by Dicer [35]. For that reason, we expected a similar inhibitory potential of exogenously administered siRNA derivatives compared to their equivalent vector based shRNAs. 11 effective shRNA sequences were randomly selected and synthesized as siRNAs to be co-transfected with the corresponding selection constructs into HEK 293 FT cells. Western blot analysis revealed that all of them were able to inhibit the TK expression from the HSV-TK/HIV fusion-mRNA constructs even at the lowest concentration used (Fig. 4a), demonstrating that our shRNA sequences could also be converted to efficient siRNAs.

It has been described that some short inhibitory sequences may exert a cytotoxic effect which was linked to the interferon (IFN) type I responses (IFN-α and IFN-β) [36], [37]. In order to test our sequences in that regard, we performed IFN-β ELISAs on HeLa cells transfected with the siRNA as described previously [38]. Even at the highest concentration used none of the above sequences induced detectable levels of IFN-β (Fig. 4b).

In addition, we investigated the inhibitory potential of the identified shRNA sequences with regard to wild-type HIV-1 infections. The corresponding 11 shRNAs were subcloned into the lentiviral vector pL and stably transduced into HIV-1 permissive HeLa P4 cells. Stable shRNA-expressing clones were infected with wt HIV-1 and the amount of the HIV-1 capsid protein p24 was determined in the culture supernatants by p24-antigen ELISA 42 h post infection. As shown in Fig. 4c, all cell clones expressing the verified shRNAs showed a significant reduction of p24 in the culture supernatant in comparison to cell clones expressing an HIV-1 irrelevant shRNA (scr). Finally, we investigated whether stable lentiviral expression of the shRNAs may have any detrimental cellular effects. As shown in Fig. 4d, none of the shRNAs impaired the division rate of the corresponding cell line compared to the controls as seen by quantitative crystal violet staining of adherent cells through four days of culture. Taken together, our data indicate that our selected shRNAs can be converted to potent siRNAs and exert a high inhibitory effect on wt HIV-1 infections, when stably expressed, both without any detectable cytotoxicity.

### Comparison of H1- and U6-promoter driven shRNA-expression

**Figure 5:**
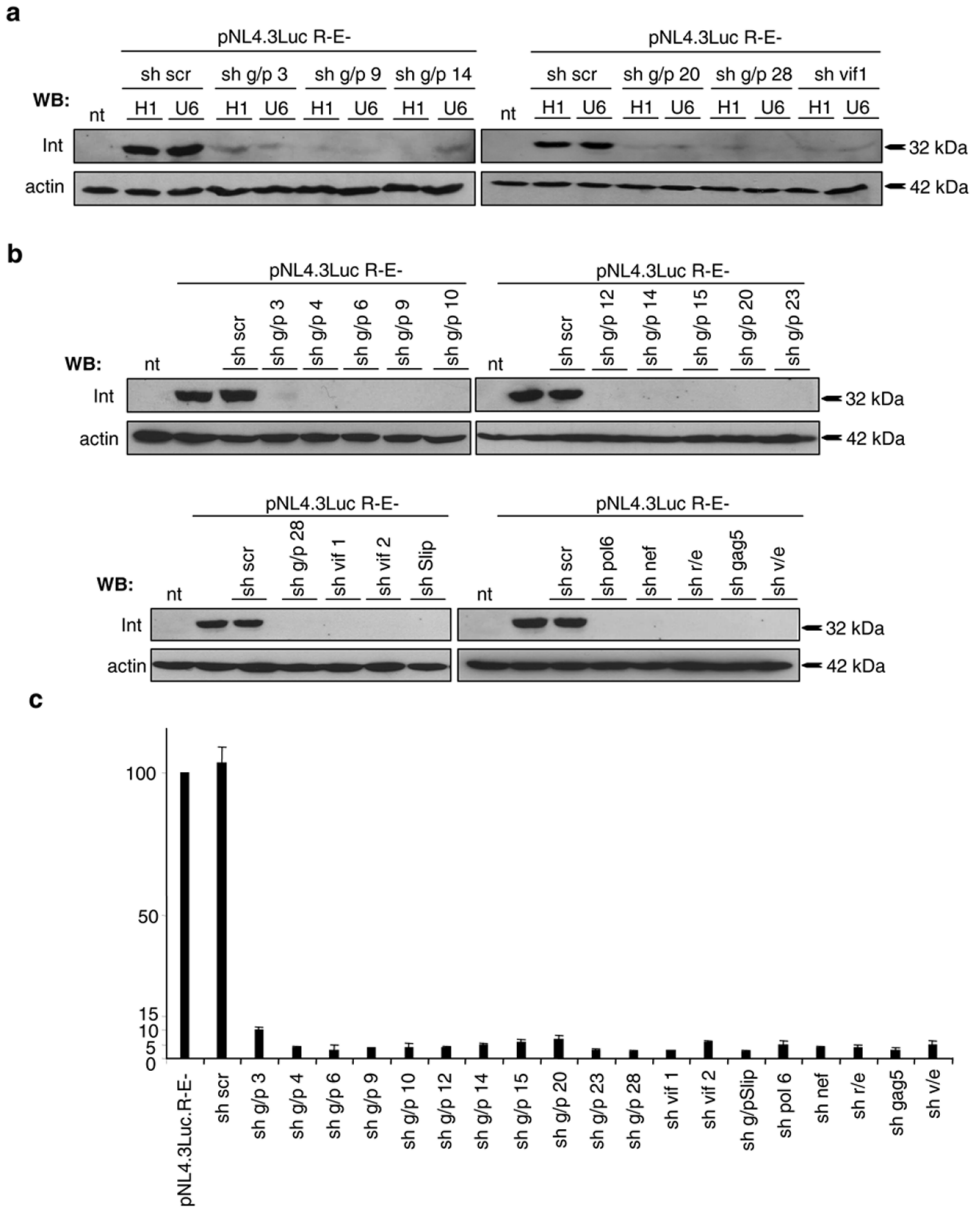
Expression of the shRNAs from different polymerase III promoters. **a**) 6 potent shRNA sequences were subcloned to allow for U6 promoter driven shRNA expression. U6 or H1 promoter expression vectors and pNL4.3LucR-E- were co-transfected into HEK 293 FT cells. Western blot analysis with a HIV-1 Integrase specific antibody 48 h p.t. indicated that the shRNAs can be efficiently expressed from both polymerase III promoters. Actin was used as loading control. **b**) **c**) Published shRNA-sequences against pol, nef, rev/env, gag and vpu/env were cloned to be expressed by the U6 promoter. These constructs as well as 14 library shRNAs and pNL4.3LucR-E- were co-transfected into HEK 293 FT cells and analysed 48 h post transfection. Western blot analysis **b**) with a HIV-1 Integrase specific antibody or **c**) luciferase assays demonstrated that the newly identified shRNAs are as potent as the published shRNAs. A scrambled shRNA (sh scr) and non-transfected cells were used as controls.

To develop an effective RNAi-based gene therapy against HIV-1, a combinatorial approach using several shRNAs expressed from different polymerase III promoters is supposed to be mandatory [39]. To this end, we tested the human U6 promoter for expression of our shRNA sequences and compared its activity against H1 promoter driven shRNA expression. Western blot analysis of integrase gene expression after co-transfection of the shRNA vectors in combination with pNL4.3R-E-Luc revealed that there is no significant difference in the efficacy of the two promoters tested (Fig. 5a).

Additionally, we compared our newly identified shRNAs with previously reported anti-HIV shRNAs. For this purpose, we generated plasmids for U6 promoter driven shRNAs against gag/pol, nef, rev and vpu/enf. The shRNAs against gag/pol [20] as well as vpu/env [18] were directed at highly conserved regions of the HIV-1 genome, whereas shNef [12] has been designed according to the siRNA algorithm provided by Dharmacon and the shRev [13] has been chosen due to the accessibility of the target mRNA. As shown in Fig. 5b, shRNAs selected from our randomized shRNA-library show comparable inhibitory potential to the published shRNA sequences. To confirm this observation, we quantified the inhibitory potential of the shRNAs by performing luciferase assays on the lysates (Fig. 5c). Together, these results demonstrate that our highly potent HIV-1 specific shRNA sequences exert comparable efficacy as to the published sequences. They could also be expressed from different promoters, which suggest them as possible candidates for combinatorial gene-therapeutic approaches against HIV-1.

## Discussion

RNA interference by means of siRNAs and shRNAs has emerged as a promising therapeutic approach against HIV-1 [40]. However, the design of highly efficient and specific siRNAs and shRNAs still remains critical. This is mainly due to the absence of a standard algorithm for the design of siRNAs [41], [42]. We generated an HIV-directed shRNA library based on the proviral DNA of HIV-1 (NL4.3), in order to systematically identify efficient HIV-1 specific shRNAs. Our study is the first application of a randomly engineered shRNA-library technology on the human immunodeficiency virus (Fig. 1). The proviral DNA of HIV-1 was fragmented using DNase I (Fig. 1a) in order to yield diverse overlapping cDNA-fragments [31], [32]. A stringent fourfold selection protocol based on positive selection via blasticidin, puromycin and EGFP fluorescence as well as negative selection by means of ganciclovir/HSV-TK in selection cell lines was applied and enabled us to enrich for cell lines expressing potent inhibitory shRNAs (Fig. 2). Besides their efficiency our sequences were devoid of any detectable cytotoxicity, which is probably also due to the stringent selection procedure (Fig. 4).

By comparing these sequences with published shRNAs, we could show that the selection of our randomized shRNA-library indeed identified efficient shRNAs with a comparable inhibitory potential (Fig. 5). Many published siRNAs or shRNAs were designed according to publicly available *in silico* algorithms. Surprisingly, none of our sequences satisfied all parameters of them. Certainly, more shRNA-sequences from randomized libraries have to be analysed to determine general sequence preferences. However, these functionally but not structurally selected shRNAs may contribute to a better understanding of the critical parameters for the design of potent siRNA and shRNA sequences. Moreover, this information could be combined with studies concerning the secondary structure and therefore the accessibility of the target-mRNA, which also influences the potential of RNAi. For this purpose Jakobsen et al. generated a sense and antisense oligonucleotide RNA-library to identify accessible target sites in the HIV-1 leader sequence, which is considerably structured, and identified five particular sites [43].

It has been shown that the activity of a lentiviral vector combining 2–3 anti-HIV shRNAs was markedly reduced with the increasing number of shRNA expression cassettes [39] based on recombination of repeat sequences in shRNA expression cassettes. We conclude, that this could be abrogated using different polymerase III promoters. Here, we could show that the shRNAs selected from our randomized library can be sufficiently expressed from different polymerase III promoters (Fig. 5). This suggests them as promising candidates for multiple shRNA approaches.

Although, retroviral shRNA gene therapy approaches showed only moderate success in the case of patients suffering from X-linked SCID [44], the durable potential of related vector systems is undeniable. Furthermore, siRNAs, e.g. against VEGF to combat AMD (age-related macula degeneration) [45], cytomegalovirus retinitis [46] and RSV infections (respiratory syncitical virus) [47] are currently being validated in clinical phase III trials, highlighting the significance of RNA-based therapeutics [48]. Thus, we liked to examine if our selected shRNAs would be suitable for a gene therapeutic approach against HIV-1. Therefore, we subcloned a range of our selected shRNAs into the lentiviral vector pL and stably transduced them into HIV-1 permissive HeLa P4 cells at a low multiplicity of infection (MOI: 0.1) to obtain low copy integrants and reduce the risk of insertional mutagenesis. After HIV-1 infection, all HeLa P4 cell lines stably expressing a HIV-1 specific shRNA showed a considerable reduction in progeny virus release in comparison to scrambeled shRNA expressing or parental cells by inhibiting the production of progeny virus.

It has been shown that more than one shRNA against HIV-1 is necessary to circumvent the generation of viral escape mutants [19], [20]. Others reported that an efficient T-cell specific delivery of three siRNAs directed against the co-receptor for HIV CCR5 and the viral vif and tat genes resulted in a significant suppression of HIV-1 infections in humanized mouse models [49]. Moreover, as an optimal strategy to prevent the emergence of escape mutants, it has been recently proposed to combine a polymerase III driven shRNA targeting the shared exon of tat and rev in combination with a TAR decoy and a chimeric VA1-ribozyme targeting human CCR5 [50]. Indeed, a pilot feasibility study using this gene therapeutic approach is already underway [48]. We suggest that e.g. a combination of our shRNAs vif1 or vif2 with the gag/pol shRNAs could target all alternatively spliced viral mRNAs and thus might provide a strong defence against HIV escape mutants (Fig. 3, Table 2). Nevertheless, a putative application of shRNA approaches would greatly benefit from the development of efficient non-integrating but stable vectors. Additionally, the present expression systems could be optimized by the use of inducible or lineage-specific promoters that drive the shRNA expression [51], [52], [53], thereby reducing possible off-target or mutagenic effects.

Recently, three whole genome screens used siRNA libraries to identify host genes critical for HIV infections [54], [55]. These screens have suggested hundreds of previously unrecognized host cell genes being involved in viral propagation and replication and hence should be considered in future approaches. Although a few HIV-specific shRNAs have been previously described, the increased numbers of potent and non-toxic shRNAs are probably needed to identify an optimal set of inhibitory sequences. This is especially true in light of the fact that shRNAs can mediate unintentional sequence-specific and –unspecific silencing of non-targeted genes. Our study clearly demonstrates that a stringently selected shRNA-library is well suited for the unbiased identification of novel potent and non-toxic shRNAs in addition to those predicted by commonly used public algorithms.
